# Conductive dendrite engineering of single-crystalline two-dimensional dielectric memristors

**DOI:** 10.1016/j.xinn.2025.100885

**Published:** 2025-03-18

**Authors:** Yu Kang, Xingyu Zhai, Quan Yang, Baoshi Qiao, Zheng Bian, Haohan Chen, Huan Hu, Yang Xu, Ming Tian, Neng Wan, Wenchao Chen, Yang Chai, Yuda Zhao, Bin Yu

**Affiliations:** 1 College of Integrated Circuits, ZJU-Hangzhou Global Scientific and Technological Innovation Center, Zhejiang University, Hangzhou 311200, China; 2 Zhejiang University - University of Illinois Urbana-Champaign Institute (ZJU-UIUC Institute), Zhejiang University, 3 Haining 14400, Hangzhou, China; 3 Department of Applied Physics, The Hong Kong Polytechnic University, Hong Kong 999077, China; 4 School of Electronics Science and Engineering, Southeast University, Nanjing 211189, China

**Keywords:** conductive dendrite engineering, single-crystalline 2D material, low-power non-volatile memristor, multi-resistance states

## Abstract

Ultralow-power non-volatile memristors are key elements in electronics. Generally, power reduction of memristors compromises data retention, a challenge known as the “power-retention dilemma,” due to the stochastic formation of conductive dendrites in resistive-switching materials. Here, we report the results of conductive dendrite engineering in single-crystalline two-dimensional (2D) dielectrics in which directional control of filamentary distribution is possible. We find that the single-vacancy density (n_SV_) of single-crystalline hexagonal boron nitride (h-BN) plays an essential role in regulating conductive dendrite growth, supported by scanning joule expansion microscopy (SJEM). With optimized n_SV_, random dendrite growth is largely limited, and electrons hop between the neighboring Ag nanoclusters in vertical channels. The corresponding model was established to probe the relationship between n_SV_ and memristor operating voltage. The conductive channel confinement in the vertical orientation contributes to long-retention non-volatile memristors with ultralow switch voltages (set: 26 mV; reset: −135 mV), excellent power efficiency (4 fW standby and a switching energy of 72 pJ) while keeping a high on/off resistance ratio of 10^8^. Even at a record-low compliance current of 10 nA, memristors retains very robust non-volatile, multiple resistive states with an operating voltage less than 120 mV (the per-transition power low as 900 pW).

## Public summary

• The variable single-vacancy density (n_SV_) is introduced during the growth of hexagonal boron nitride.
• The relationship between n_SV_ and operating voltage was established experimentally, along with a corresponding model.
• The n_SV_ determines the performance by regulating dendrites, as seen via scanning joule expansion microscopy.
• Memristors exhibit record performance with a set voltage of 26 mV and excellent power efficiency.
• Memristors with non-volatile and multi-resistive states are achieved with a retention time of over 10 years.

## Graphical abstract

**Figure:**
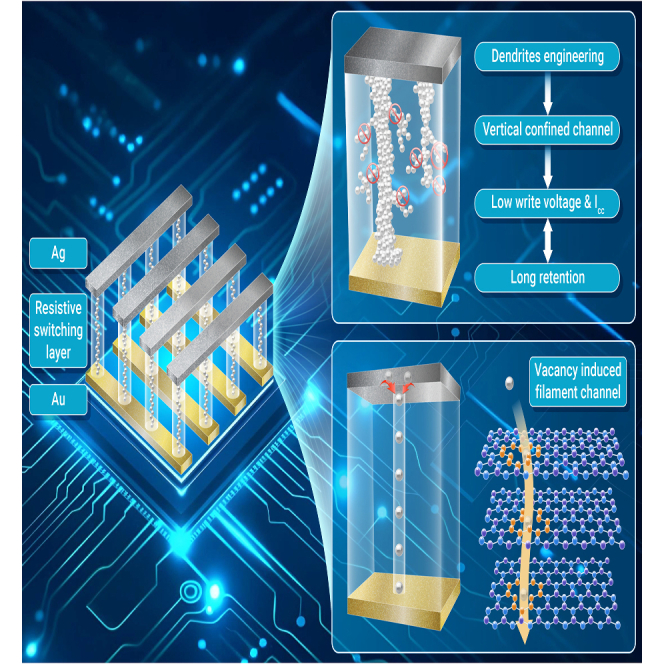

## Introduction

**Figure 1:**
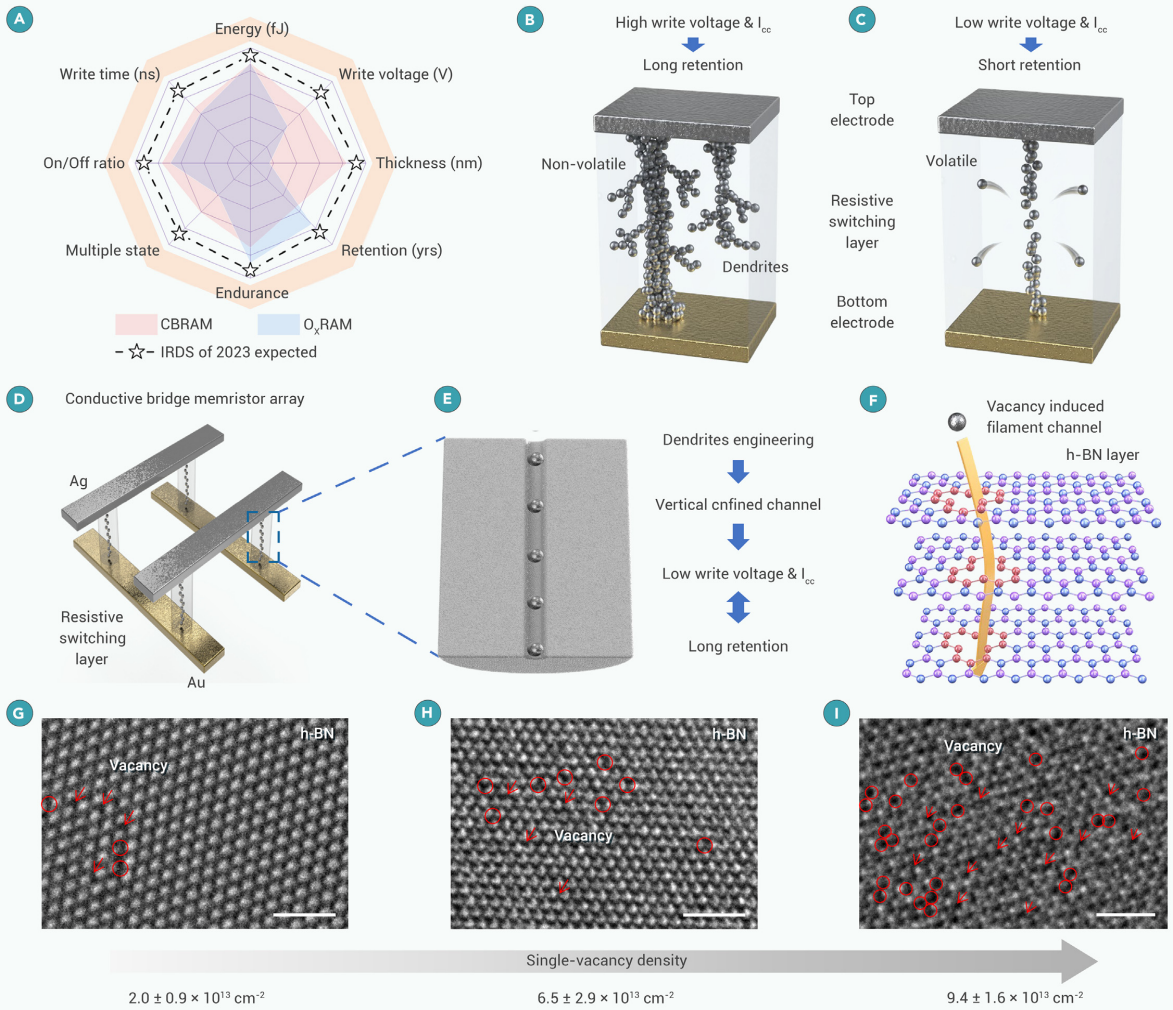
Single-crystalline h-BN with tunable defect density (A) Benchmarks of two types of memristors (resistive random-access memory [RRAM] as in the International Roadmap for Devices and Systems (IRDS) prediction). (B and C) Schematic illustration of power-retention dilemma in memristor. (D) Schematic of a conductive bridge memristor array. (E) Illustration of a nanosized filament in confined channel. Ag ions migrate through the predefined nanosized pathway, inhibiting the random growth of the conductive filament. (F) Schematic of Ag atom passing through the vertically aligned filament path in h-BN. When the defects in h-BN are aligned to the vertical direction, Ag atoms pass through the shortest distance between two electrodes. (G–I) Single-vacancy defects in single-crystalline h-BN observed in HRTEM (accelerating voltage: 80 keV). The SV defects are highlighted by red circles and arrows. The scale bar represents 1 nm. The SV density increases from 2.0 × 10^13^ to 9.4 × 10^13^ cm^−2^.

Memristors have found a range of applications in next-generation electronics,^1^ boosting data-processing efficiency with emergent paradigms such as computation-in-memory^2^^,^^3^ and neuromorphic computing.^4^^,^^5^ The sustainable development of future hardware demands energy-efficient, non-volatile memristors with aggressively scaled voltages and currents.^6^^,^^7^ However, there is a fundamental conflict between power and non-volatility, known as the power-retention dilemma, resultant from the stochastic spatial formation and rupture of conductive dendrites in resistive-switching (RS) media, most of which are amorphous (Figures 1A and 1B).^8^ Under a low set voltage and/or small compliance current (I_cc_), conductive filaments are unstable due to high surface free energy, leading to undesired volatile behaviors (Figure 1C). To implement ultra-scaled memristors operating with minimized power and excellent retention, it is compulsory to break the limit of the conductive mechanism via engineering filamentary growth dynamics.^9^^,^^10^

Conductive dendrites, the spatial profile of randomly formed conductive filaments, play an essential role in ruling the uniformity, stability, and power efficiency of memristors. Regulated formation of conductive dendrites results in self-confined vertical channels that minimize excess energy loss for ion diffusion, which is necessary for ultralow-power operations (while retaining structural stability, or non-volatility). Memristors with shrunken electrode areas and/or RS medium thickness have displayed reduced filament numbers or size.^11^^,^^12^^,^^13^ However, the devices show considerably increased switch voltages and small on/off resistance ratios as the compliance current is lowered.^14^^,^^15^ Most devices exhibit a transition from non-volatility to volatility due to unstable filaments.^16^^,^^17^ The bottleneck can be overcome via predefined channels in RS or new transport mechanisms.^18^^,^^19^^,^^20^ Recently, memristors with filamentary channels resulting from helium ion irradiation^21^ or a grain-boundary-assisted mechanism^22^ were reported. Two-dimensional (2D) materials have been explored for memristors.^23^ Different from traditional amorphous thin films, the defects and ionic activities in single-crystal 2D layers can be controlled by an electric field.^24^ Leveraging the unique features of single-crystalline 2D dielectrics for conductive dendrite engineering is expected to resolve the power-retention dilemma of ultra-scaled memristors. However, it has not yet been researched to date.

In this article, we report robust, non-volatile memristors with excellent power efficiency by engineering a dendrite profile in single-crystalline 2D dielectric hexagonal boron nitride (h-BN). Variable single-vacancy (SV) density (n_SV_) is introduced during h-BN growth. Under an electric field, the vacancies are prone to migrating into a vertically aligned path during the forming phase,^25^ and limited dendrite growth results in localized channels in a vertical orientation. In the low resistance state (LRS), electrons hop between the neighboring Ag clusters. The n_SV_ regulates the dendrite profile, supported by the results of scanning joule expansion microscopy (SJEM). The corresponding model has been established to probe the relationship between the n_SV_ and operating voltage. The dendrite-free profile with the optimized n_SV_ leads to the shortest conductive path perpendicular to electrodes, resulting in minimized power. Our demonstrated memristor shows stable, non-volatile RS with operating voltages as low as 26 (set) and −135 (reset) mV and excellent power efficiency (4 fW standby and a switching energy of 72 pJ) while keeping a high on/off resistance ratio of 10^8^. The device retains non-volatile, multi-resistive-state behavior with an operating voltage of less than 120 mV and an I_cc_ down to 10 nA (900 pW per transition), showing the best record of low-power memristors.

## Materials and methods

### Growth of single-crystalline h-BN

Carbon was introduced as a dopant during crystal growth to regulate the SV defect in h-BN. A cylindrical h-BN crucible was filled with h-BN powder and carbon powder. Ni-Cr alloy was then added to the h-BN powder. The alloy, the h-BN powder, and the pure carbon powder were weighed in a ratio in a series of experiments. To reduce the residual oxygen gas in the furnace, the furnace was evacuated three to five times to a vacuum of 10 Pa and then purged with nitrogen before ramping to high temperature. The sample was heated to 1,450°C at a rate of 6°C/min. After being held at 1,450°C for 12 h, the system was slowly cooled to 1,200°C at a rate of 4°C/h. The sample was then allowed to naturally cool to room temperature. Throughout the growth period, the flow rate of N_2_ gas was maintained at ∼100 sccm. By the method, the different single vacancy density (n_SV_) was introduced in the single-crystalline material h-BN. The fabricated h-BN single crystals were mechanically exfoliated by Scotch tape for material characterization and device fabrication.

### Fabrication of memristor

The bottom electrode of Cr/Au (5/35 nm) with an average width of ∼3 μm was patterned on 285 nm SiO_2_/Si substrates using standard photolithography and sputtering. The mechanically exfoliated h-BN flakes with a thickness of 6 nm were transferred onto the bottom electrode with the help of polydimethylsiloxane (PDMS). Then, the van der Waals (vdW) Ag electrode was transferred using a PDMS stamp onto the h-BN flake as the top electrode. It is worth noting that the top electrode Ag was dry transferred onto the h-BN layers to form a vdW contact, which can avoid the introduction of defects during the metal deposition process and preserve the high-quality RS layer with the predefined defect type/density.^26^^,^^27^^,^^28^

### vdW electrodes

First, Poly(methyl methacrylate (PMMA) was spin coated onto a silicon wafer at 3,000 rpm for 20 s, then it was rapidly dried using a hot plate at 150°C for 20 min. Polyimide (PI) solution was spin coated on top of the PMMA layer at 600 rpm for 20 s and 4,500 rpm for 40 s then baked at 220°C for 20 min. The PI layer had weak adhesion to the PMMA layer. It could be mechanically removed using a PDMS stamp with PMMA as a sacrificial layer. The PDMS solution (DOW CORNING 184) was used with a standard curing process to get the PDMS stamp. The 40 nm Ag top electrode was patterned onto the substrate with a PI thin film using standard photolithography and magnetron sputtering. The width of the top Ag electrode was 3 μm. The Ag vdW electrode with the PI layer was released from the silicon wafer with the PDMS stamp, and the PI layer could be easily removed through dry etching.

### Device and material characterization

The electrical measurements of I-V sweeping were conducted using a Keithley 2450 source meter. Prior to temperature-dependent performances, the memristor was switched to an LRS state at room temperature. Then, the I-V characteristics of the memristor at various temperatures in the LRS state were obtained after corresponding temperature stabilization. The pulse test was characterized using a probe station connected to a Keithley semiconductor analyzer 4200-SCS equipped with pulse measuring units. The bias was applied to the Ag electrode, and the Au bottom was grounded. Spherical aberration-corrected transmission electron microscopy (AC-TEM; FEI Titan G2 60-300), where both spherical and chromatic aberrations are corrected, was used to characterize the crystal structure of the h-BN sheet. The samples have been observed with AC-TEM at an accelerating voltage of 80 keV to minimize damage to the sample. An atomic force microscope (AFM; Bruker Dimension ICON) was used to identify the thickness of the h-BN. A scanning electron microscope (SEM; Zeiss Sigma300) was used to identify the structure of the h-BN memristor. The cross-sectional TEM samples were fabricated using the *in situ* focused ion beam (FIB) lift-out technique on a Thermo Scientific Helios G4 HX FIB/SEM, and the sample was imaged with FEI Titan Themis G3 60-300.

### SJEM measurements

In the device circuit, a 35 kHz square wave with a 50% duty cycle, 0.5 V amplitude, and 0.25 V offset was generated by a signal generator (SIGLENT SDG2122X) and applied to the memristor to produce modulated joule-heat-induced deformation. The tiny thermal expansion of surface imaging was performed using an AFM contact mode probe (Olympus AC240TSA-R3) mounted on an MFP-3D-Origin+ device. Surface displacement images were analyzed by a signal recovery OE1022 lock-in amplifier (Sine Scientific). The measurement can be performed in the steady state, where a constant voltage is applied to the device and the AFM probe scans the electrode surface to obtain the thermal expansion height.

### RS characteristic modeling

We develop a compact model to explain the influence of SV defect densities on the RS behavior of the memristor, which is applicable when n_SV_ is not enough to form large-size vacancies. The proposed model consists of three modules: a silver ion migration module, a self-heating effect module, and a current conduction module (details in Note S2). The silver ion migration module describes the dynamic percentage of Ag-occupied vacancies (P, which is defined as the number of occupied vacancies divided by the total number of vacancies) using the ion hopping equation, considering the variation of Ag ions’ hopping distances. The different conductive mechanisms in the high resistance state (HRS) and LRS are considered in the current conduction module.

## Results and discussion

### Tunable vacancy density in 2D single-crystal h-BN

Figure 1D shows the schematic of a conductive bridge memristor using Ag and Au as two electrodes. The Ag electrode (active) can be oxidized by applying voltage, and the Ag ions migrate through the RS layer under a vertical electric field to the Au electrode (inert). To minimize power, dendrite growth should be regulated. Because the movement of Ag ions is random under the action of an electric field, especially if there is no predefined path in the dielectric layer. To break this limit, it is proposed to trap the Ag filament in a vertically aligned nanochannel (Figure 1E). In the ideal condition, the predefined channel traps Ag ions, impeding conductive dendrite growth. In our experiment, single-crystalline h-BN is used as the RS layer with intentionally introduced vacancies (Figures 1F and S1). Compared with metal oxides and chemical vapor deposition (CVD)-grown h-BN, single-crystalline h-BN exhibits the advantages of limited defects and tunable defect density. During the formation process, the vacancy defects in h-BN can form a nanosized channel driven by the electric field,^29^ which facilitates confined filament growth. The vacancy density is a critical parameter to control the spatial profile of the channel (e.g., size and verticalness).

The atmospheric-pressure metal-flux-based fusion method was used to prepare single-crystalline h-BN with the tunable defect density by adjusting the weight ratio of carbon powder in the fusion alloy. Figures 1G–1I show the representative high-resolution TEM (HRTEM) images of the grown h-BN monolayers with the high-quality single-crystalline structure. The tunable defect densities were confirmed by statistically analyzing more than 30 HRTEM images (Figures S2A–S2C). The density of SV defects (n_SV_) can be tuned from 2.0 × 10^13^ to 9.4 × 10^13^ cm^−2^. The high-pressure and high-temperature (HPHT) method was used to prepare the highest-quality h-BN single crystals with the lowest SV defect densities (n_SV_ = 1.0 × 10^10^ cm^−2^). The HRTEM images display indiscernible defects in the 5 × 5 nm region (Figure S3). For comparison, CVD-grown h-BN displays complex defect types and large-size nanopores (Figure S4), leading to the random and uncontrollable growth of Ag filaments.

### Vacancy-density-dependent memristive behavior

**Figure 2:**
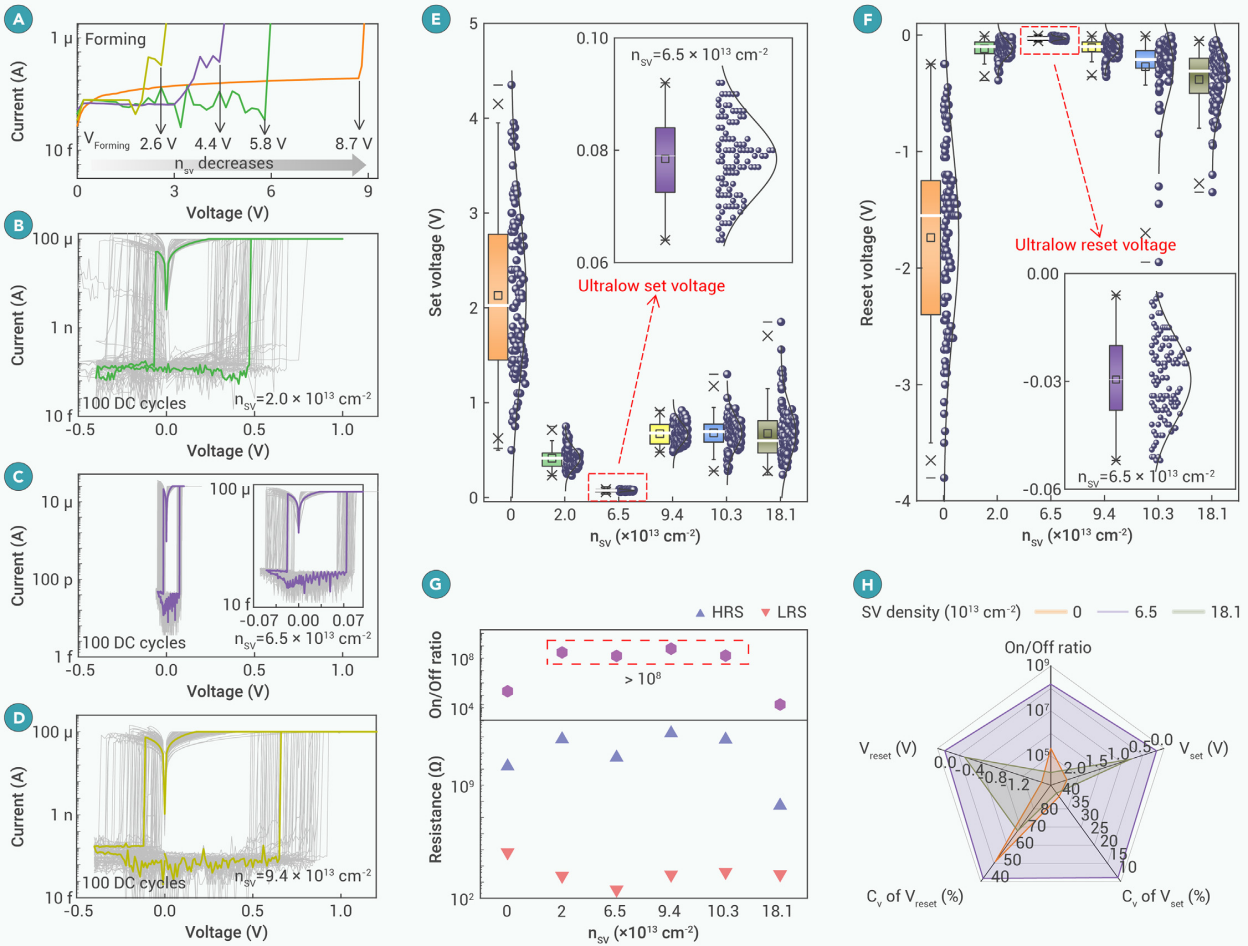
Resistive switching of h-BN memristor with varying SV density (A) I-V curves during the forming process in 3 × 3 μm Ag/h-BN/Au memristor with tunable SV density. (B–D) Non-volatile, bipolar RS behavior in single-crystalline h-BN with different SV densities when I_cc_ is 0.1 mA. 100 consecutive I-V curves were collected, and the representative curve is highlighted. (E and F) Statistical analysis of set/reset voltages with different SV densities. The inset shows the distribution of set/reset voltages with n_SV_ = 6.5 × 10^13^ cm^−2^. (G) The summary of HRS and LRS resistances from 100 consecutive direct current (DC) I-V curves and the on/off ratio dependent on n_SV_. (H) Benchmark of five device metrics between h-BN memristor with three SV densities. C_V_, coefficient of variation; C_V_, σ/μ, where σ and μ are the standard deviation and mean value of operating voltage, respectively.

To explore the relationship between the defect density in h-BN and memristor performance, single-crystalline h-BN with defect densities ranging from 1.0 × 10^10^ to 9.4 × 10^13^ cm^−2^ are used as the RS layer. The conductive bridge memristor has a sandwiched Ag/h-BN/Au structure (see SEM and TEM images in Figures S5A and S5B), and the thickness of the h-BN flake is ∼6 nm (Figure S5C). For each defect density, we fabricated at least 5 devices, and the typical behaviors are shown in Figures 2 and S6–S8.

The I-V curves of single-crystalline memristor with different SV defect densities are shown in Figure 2A under an I_cc_ of 0.1 mA. When the RS medium is almost defect free (n_SV_ = ∼1.0 × 10^10^ cm^−2^), it requires relatively high energy to induce a soft breakdown of h-BN and randomly create a conductive path, leading to an ultrahigh forming voltage of 8.7 V. As the defect density increases, the forming voltage gradually decreases. The single-crystalline h-BN with moderate n_SV_ (n_SV_ ranges from 2.0 to 9.4 × 10^13^ cm^−2^) displays a forming voltage of 2.6–5.8 V, indicating that the conductive paths are prone to forming at the defective regions. A higher defect density facilitates the formation of conductive paths. Therefore, the introduction of defects in single-crystalline h-BN can lower the forming voltage and mitigate the stochastic formation of conductive paths.

After the formation of the initial conductive path, all devices show non-volatile switching with an I_cc_ of 0.1 mA (Figures 2B–2D). When the intrinsic defect density of the h-BN is 1.0 × 10^10^ cm^−2^, the operating (set and reset) voltages are at a maximum, and the I-V curves are unstable, with large cycle-to-cycle variation (Figure S6) due to the formation of random channels during the formation. The single-crystalline h-BN with intentionally introduced defects (n_SV_ from 2.0 to 9.4 × 10^13^ cm^−2^) can effectively confine the conductive path. The memristors display stable I-V switching cycles with operating voltages of less than ±1 V (Figures 2E and 2F). With the increasing SV defect density in the material, the operating voltages first decrease and then increase, showing a “U-shaped” feature (Figure 2E). An ultralow set voltage of ∼73 mV is observed with an n_SV_ of 6.5 × 10^13^ cm^−2^ (Figure 2C). The distribution of operating voltages follows a symmetric normal pattern, indicating stable and reliable non-volatile RS behavior. Statistical analysis of 9 single-crystalline h-BN memristors with an n_SV_ of 6.5 × 10^13^ cm^−2^ (Figure S9) shows that the averaged set and reset voltages are 65 and −14 mV, respectively, suggesting small device-to-device variation. The measured RS ratio is over 10^8^ (Figure 2G). The dependence of thickness on the electrical performance of single-crystalline h-BN-based memristors with an n_SV_ of 6.5 × 10^13^ cm^−2^ has also been studied (Figure S10), and the dielectric layer of 6 nm shows the best ability for dendrite engineering.

Figure 2H summarizes the figures of merit (FOMs) of the h-BN memristor, including the set and reset voltages (and their variations) and the on/off switching ratio. The memristor with an n_SV_ of 6.5 × 10^13^ cm^−2^ displays the lowest set (73 mV) and reset (−29 mV) voltages with the smallest coefficients of variation (C_v_; 9.53% for set and 34.93% for reset) and preserves a high on/off switching ratio (10^8^). The results demonstrate that engineering SV defects in the single-crystalline h-BN layer plays an essential role in achieving optimal memristive performance.

### Conductive dendrite engineering in single-crystalline h-BN

**Figure 3:**
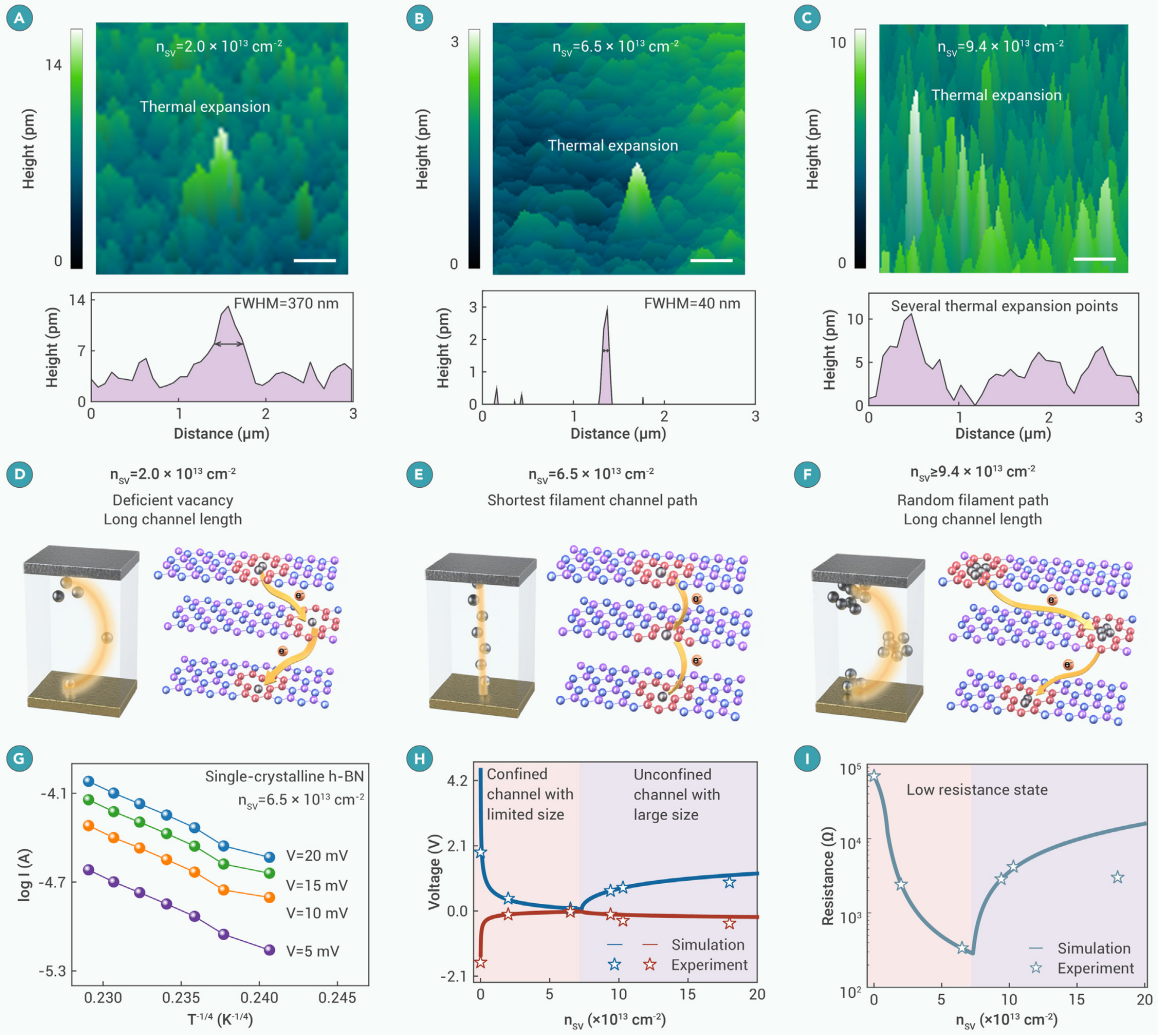
Conductive dendrite engineering in single-crystalline h-BN (A–C) SJEM images of h-BN memristor with n_SV_ from 2.0 × 10^13^ to 9.4 × 10^13^ cm^−2^ (3 × 3 μm active device area; scale bar represents 500 nm) and the corresponding height profile. (D and E) Schematic diagrams of the filament path in single-crystalline h-BN memristor with n_SV_ = 2.0 × 10^13^ and 6.5 × 10^13^ cm^−2^. (F) Schematic diagram of the filament path with an n_SV_ greater than 9.4 × 10^13^ cm^−2^, indicating the formation of a large-size channel. (G) Plot of log(I) versus T^−1/4^ for the h-BN memristor (n_SV_ = 6.5 × 10^13^ cm^−2^) at varying temperatures. (H) Simulated set/reset voltages of single-crystalline h-BN memristor dependent on n_SV_. The experimental results are marked on the curves. (I) Relationship between LRS resistance and n_SV_. The experimental results are marked on the curves.

SJEM^30^ was used to characterize the size and spatial distribution of the filament channel in single-crystalline h-BN with different SV densities (details in Note S1 and Figure S11). Several approaches of conventional conductive AFM and TEM can visualize conductive paths, but it causes damage to the device structure. SJEM is a non-destructive surface displacement imaging technique that monitors the local temperature by measuring the thermal expansion of the sample due to joule heating,^31^^,^^32^ which accurately detects the distribution of conductive paths in the memristors with ultrahigh spatial resolution (∼10 nm). Figures 3A–3C display the thermal expansion mapping of h-BN memristors with n_SV_ of 2.0, 6.5, and 9.4 × 10^13^ cm^−2^, respectively. In the n_SV_ = 2.0 × 10^13^ cm^−2^ sample, the spatial distribution of the thermal expansion point is around 370 nm (full width at half maximum [FWHM]). In comparison, the thermal expansion points of the n_SV_ = 6.5 × 10^13^ cm^−2^ sample are highly localized in a circular region, with an FWHM of 40 nm. The results provide evidence of a highly confined filament channel in the latter, leading to inhibited dendrite growth. The SJEM on the n_SV_ = 9.4 × 10^13^ cm^−2^ sample shows several thermal expansion points distributed over the 3 × 3 μm area, indicating relatively random filament growth. We believe that in the formation process, the initial SV densities in h-BN determine the spatial profiles of the filament path, including the filament channel size, length, and verticalness. An optimized SV defect density (n_SV_ = 6.5 × 10^13^ cm^−2^) results in the shortest Ag drift length perpendicular to electrodes with negligible dendrite growth (Figure 3E). In this condition, the set and reset voltages are the smallest to reach the threshold electric field (*E*_th_ = V_set_/channel length) for RS. In contrast, the tilted filament channels (n_SV_ = 2.0 × 10^13^ and 9.4 × 10^13^ cm^−2^) lead to a reduced electric field (Figures 3D and 3F), and the set voltage has to be increased. It is worth noting that when n_SV_ exceeds 6.5 × 10^13^ cm^−2^, large-size vacancies are easily created during the formation. They are difficult to move, displaying a random distribution consistent with the SJEM results.

The conduction mechanism is analyzed by the double logarithmic I-V curves (Figure S12). In the HRS, the slope remains nearly zero as the abrupt current increases (Figures S12A–S12C). In comparison, the CVD-grown h-BN memristor (Figure S12D) exhibits the classical thermionic emission (low bias) and space-charge limited current (high bias). The results demonstrate that the device displays an unusual switching mechanism, which is related to the highly confined filament channel. In the LRS, the temperature-dependent electrical measurement was conducted. In the log(I) versus T^−1/4^ curves (Figure 3G), the current increases with rising temperature, indicating a negative temperature coefficient, which is different from the traditional metallic filament memristors.^33^^,^^34^^,^^35^ This suggests that the LRS conduction in single-crystalline h-BN follows the electron hopping behavior.

We established a physical model to verify the filament dynamics in single-crystalline h-BN memristors (details in Note 2). The simulated DC I-V behavior for all n_SV_ concentrations is in good agreement with the experimental data, as depicted in Figure S13. As n_SV_ increases from 1.0 × 10^10^ to 6.5 × 10^13^ cm^−2^ (pink region, Figures 3H and 3I), the extracted set/reset voltages decrease, and the LRS resistance lowers from 6.93 × 10^4^ to 3.37 × 10^2^ Ω. This is because the length of the filament path is inversely proportional to n_SV_. A shorter length leads to a boosted electric field and a higher migration probability of Ag ions as well. Increasing the Ag ion concentration in the conductive path results in a shortened hopping distance and, hence, lowered LRS resistance. When n_SV_ exceeds 6.5 × 10^13^ cm^−2^ (violet region, Figures 3H and 3I), both operation voltages and LRS resistance slightly increase and then saturate. The filament channel length increases after the formation of immovable large-size vacancies, resulting in higher operating voltages and LRS resistance. The Ag atoms can hardly be trapped in the large-size vacancy, causing degraded device stability due to filament stochasticity. Therefore, an optimized n_SV_ inhibits the formation of conductive dendrites, improving the power efficiency and reliability of the memristor.

### Ultralow-voltage memristors

**Figure 4:**
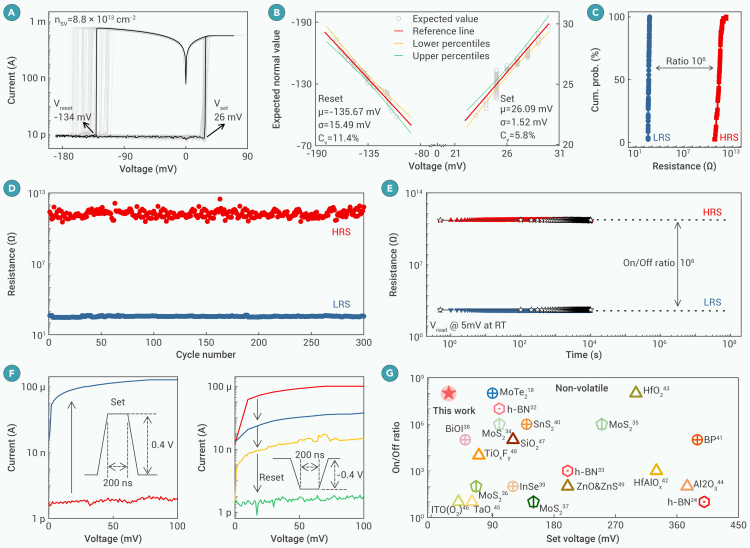
Ultralow operating voltages in non-volatile h-BN-based memristor with n_SV_ = 8.8 × 10^13^ cm^−2^ (A) Representative I-V characteristics during 80 consecutive cycles in 3 × 3 μm Ag/h-BN/Au non-volatile memristor with I_cc_ of 0.1 mA. (B) Quantile-quantile plots curve of the set and reset voltages. The experimental value of the operating voltage is consistent with the expected value, indicating that the operating voltage has a normal distribution. (C) Cumulative probability distribution of the HRS and LRS. (D) Endurance performance of h-BN memristor. (E) Retention performance of the h-BN memristor at a read voltage of 5 mV. The dotted lines connected by the five-pointed stars represent the resistance of the memristor every 100 s, indicating that there is no loss in the resistance change during the test. (F) Pulsed set/reset characterization. The memristor switches from an HRS to an LRS after applying a pulsed set voltage of 0.4 V for 200 ns. (G) Comparison of the on/off ratios versus set voltages among non-volatile bipolar memristors in the sub-450 mV regime. Our memristor shows an ultralow voltage while maintaining a larger on/off ratio among the reported memristors (different patterns represent different types of material: hexagonal, h-BN; pentagonal, MoS_2_; circular, other 2D materials; and triangle, 3D oxide).

To statistically understand the memristor performance, we measured 10 devices at the optimal SV defect density (considering fluctuation, n_SV_ = 6.5 ± 2.9 × 10^13^ cm^−2^) and found that the set voltage can be lowed to 26 mV, which corresponds to an n_SV_ of 8.8 × 10^13^ cm^−2^, as extracted from the theoretical model (Figure 3H). The I-V curves are obtained by sweeping the voltage across the top/bottom electrodes for 80 consecutive cycles on a 3 × 3 μm active area. The memristor exhibits reproducible, non-volatile, bipolar RS behavior with remarkable uniformity (Figure 4A) (I_cc_ = 0.1 mA). The benchmarked C_v_ values are 5.8% and 11.4% for the set and reset voltages, respectively. The data within the 95% confidence interval for the operation voltage indicate that the non-volatile behavior is stable and repeatable (Figure 4B). Since HRS and LRS resistance distributions are about 8 orders of magnitude apart, the two distinct states are easy observe (Figure 4C). The standby power (P_Standby_ = I_HRS_ × V_READ_) reaches as low as 4 fW with an HRS current of 4.93 pA (read voltage: 1 mV). The cycle-to-cycle endurance (Figure 4D) exhibits temporal uniformity, indicating robust, non-volatile behavior. Notably, the device also displays excellent retention (Figure 4E). The decay rates of LRS and HRS resistances over time are negligible for 10^4^ s.

In addition to the DC test, pulsed set/reset measurements were conducted (Figure 4F). The device switches directly from an HRS to an LRS by applying a set voltage pulse of 0.4 V for 200 ns. A reset pulse of −0.4 V for 600 ns performs the transition from an LRS to an HRS. A higher reverse voltage (−0.7 V) for 200 ns also induces the switching from an LRS to an HRS (Figure S14). An ultralow energy (∼72 pJ/bit) and a fast-switching time (∼100 ns) are achieved. To benchmark the device performance, Figure 4G compares the on/off ratios and set voltages among those recently published non-volatile memristors. Operating at an ultralow voltage with a high switching ratio, our device outperforms other 2D material memristors^18^^,^^22^^,^^35^^,^^36^^,^^37^^,^^38^^,^^39^^,^^40^^,^^41^^,^^42^^,^^43^^,^^44^ and oxide-based memristors.^45^^,^^46^^,^^47^^,^^48^^,^^49^^,^^50^^,^^51^^,^^52^

### Non-volatile multiple-state switching at ultralow I_cc_

**Figure 5:**
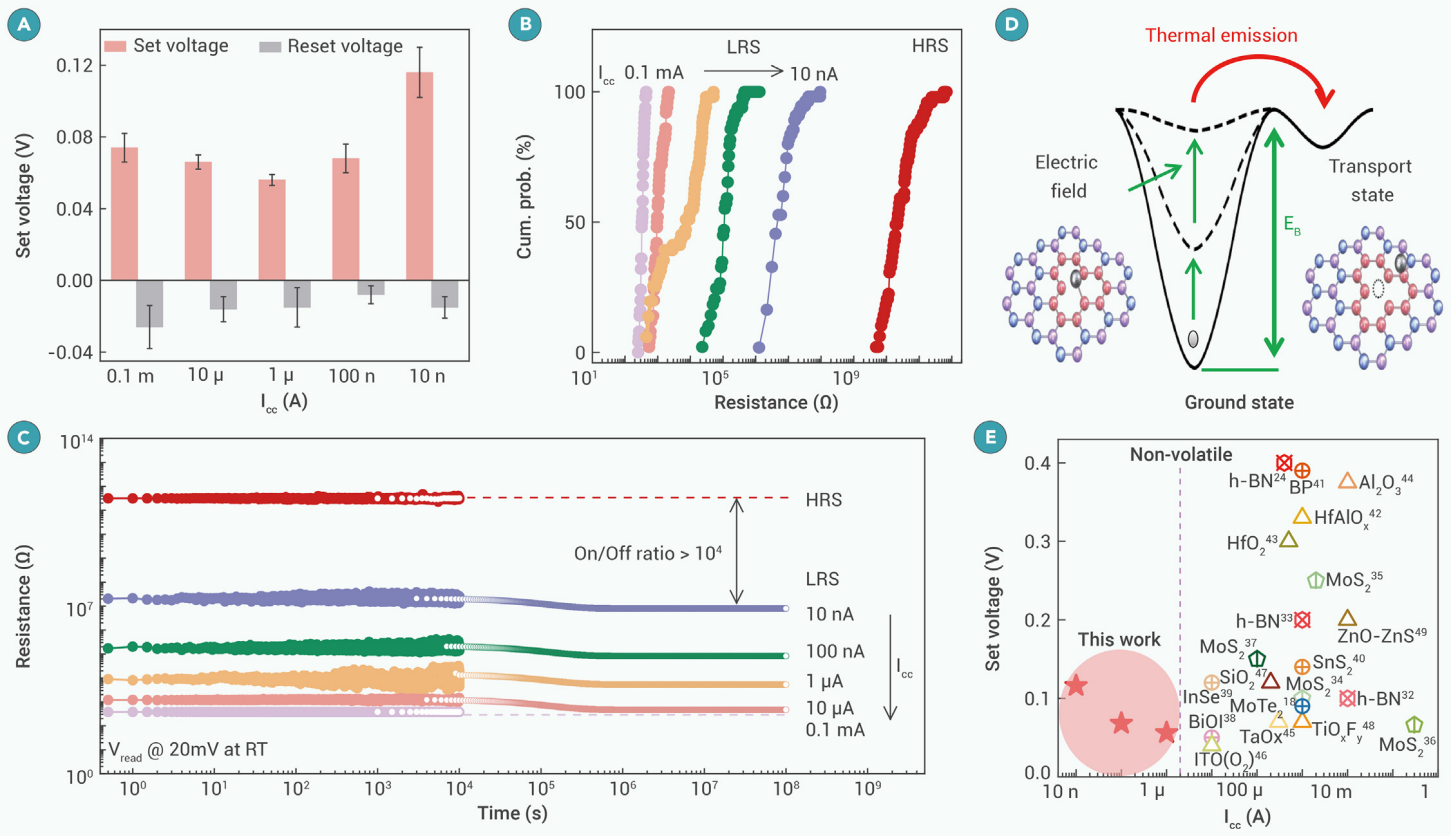
Preserving non-volatility under ultralow I_cc_ (A) The memristor operation voltages display a slight variation under various I_cc_ from 0.1 mA to 10 nA. (B) Cumulative probability distribution of HRS and LRS resistances fitted from 50 DC I-V cycles under varied I_cc_ (10 nA–0.1 mA) with n_SV_ = 6.5 × 10^13^ cm^−2^. (C) Retention performance at a read voltage of 20 mV. It is worth nothing that we used experimental data to predict the resistive change in ultra-long standby times with I_cc_ from 10 μA to 10 nA. The resistance first decreases and then tends to stabilize after a short period. The resistive state is projected to remain stable for ultra-long retention times. (D) Schematics of the interaction between Ag and h-BN filament channel. (E) Scatterplot comparing the set voltage versus I_cc_ among non-volatile bipolar memristors.

In filament-based memristors, maintaining non-volatile switching with an ultralow I_cc_ is a critical challenge. When the I_cc_ is low, the filament is weak and self-dissolving, leading to volatile switching. Prior efforts have shown that non-volatile switching behavior occurs in small device areas of 0.4 × 0.4 μm^2^ at low I_cc_.^53^ Here, we achieve a robust, non-volatile multiple state with an n_SV_ of 6.5 × 10^13^ cm^−2^ under different I_cc_ through dendrite engineering. Figures S15A–S15E show the I-V curves under I_cc_ from 0.1 mA to 10 nA with an ultralow per-transition power of 900 pW (P_per-transition_ = I_cc_ × V_set_). The set and reset voltages are kept below 0.12 and −0.03 V, respectively, displaying a slight variation, which is dependent on I_cc_ (Figure 5A). An insignificant overlap is observed between the HRS and LRS under all I_cc_ (Figure 5B), indicating that the two states can be distinguished. In addition, the device maintains an RS ratio of 10^4^, even with an I_cc_ of 10 nA (Figure S15F). The LRS resistance increases by two orders of magnitude when the I_cc_ reduces from 100 to 10 nA, which implies that the electron hopping distance greatly increases. It is worth noting that the resistances and switching ratios at different I_cc_ show robust retention (reading voltage: 20 mV) (Figure 5C). The HRS resistance displays negligible degradation within 10^4^ s, while the LRS resistance slightly decreases due to the redistribution of Ag clusters. By extrapolating the retention curves, we deduce that the six resistance states, including one HRS state and five LRS states, can be distinguished for ultra-long standby times (simulation details in Note S3).

The excellent retention can be explained by the trapping of Ag nanoclusters in the predefined filament pathways, which results in a strong interaction between the Ag atoms and the neighboring h-BN atoms. The self-diffusion of Ag atoms from the ground state into the mobile transport state is very difficult (Figure 5D). The diffusion potential and the Gibbs-Thomson potential of Ag are insufficient to overcome the high energy barrier E_B_ (−5.01 eV).^54^ It guarantees non-volatile memristor operations under ultralow I_cc_, which is a unique feature of our devices. Therefore, we are able to scale down the voltage and current while maintaining excellent data retention, solving the power-retention dilemma. Figure 5E shows the benchmarking of our non-volatile memristor. The ultralow set voltage of 116 mV with an I_cc_ of 10 nA is one of the lowest values for non-volatile memristors ever reported.^18^^,^^22^^,^^35^^,^^36^^,^^37^^,^^38^^,^^39^^,^^40^^,^^41^^,^^42^^,^^43^^,^^44^^,^^45^^,^^46^^,^^47^^,^^48^^,^^49^^,^^50^^,^^51^^,^^52^

## Conclusion

To tackle the well-known power-retention dilemma in RS-based memristors, we took the approach of leveraging the advantage of single-crystalline 2D dielectrics and the microscopic material engineering strategy through conductive dendrite profile regulation. By introducing a vertically aligned conductive nanochannel in h-BN with an optimized n_SV_, the random formation of conductive filaments is restricted, resulting in the superb performance of the memristor. The set voltages are reduced to as low as 26 mV, the lowest operation voltage on record for a non-volatile memristor, while retaining a high on/off resistance ratio of 10^8^ and long retention. The power efficiency is significantly boosted (900 pW per transition, 4 fW standby, and a switching energy of 72 pJ). Further, the trapping of Ag nanoclusters in a self-confined channel leads to robust non-volatility and a multi-resistance state at an I_cc_ down to 10 nA with a projected long retention. Potential applications include ultra-dense memories, neuromorphic computing, bio-compatible interfaces, and various edge sensing arrays based on ultralow-voltage/current memristors, and memristors' derivative structures combined with CMOS technology. However, the commercial application of low-voltage memristors still faces many impediments, which is an issue for further research.

## Resource availability

### Materials availability

All unique/stable reagents generated in this study are available from the lead contact with a completed materials transfer agreement.

### Data and code availability

The data that support the findings of this study are available from the corresponding author upon reasonable request.

## Funding and acknowledgments

We are thankful for the support from 10.13039/501100001809NSFC (92264106, 62090034, 62104214, 62122067, and 62261160574), the Research Grant Council of Hong Kong (CRS_PolyU502/22), the National Key R&D Program (2022YFA1204303), the NSFC of Zhejiang Province (DT23F0401 and DT23F040008), and the Young Elite Scientists Sponsorship Program by CAST (2021QNRC001). We also thank the ZJU Micro-Nano Fabrication Center and ZJU-Hangzhou Global Scientific and Technological Innovation Center for support.

## Author contributions

B.Y., Y.Z., W.C., and Y.C. supervised the project. Y.K., Y.Z., and B.Y. designed the experiments. Y.K. carried out the device fabrication and the electrical characterization. X.Z. and W.C. provided theoretical analysis and modeling. Q.Y. helped with data analysis. B.Q., Y.K., and H.H. carried out SJEM tests and analysis. Z.B. and H.C. carried out AFM tests and analysis. M.T. and N.W. contributed to the material growth. Y.K. carried out the TEM measurements, and N.W. helped with data analysis. Y.K. wrote the manuscript. Y.X. and Y.Z. helped with paper writing. Y.C. improved the section on device applications. All authors contributed to the manuscript and approved the final version.

## Declaration of interests

The authors declare no competing interests.

## Supplemental information

Document S1. Figures S1–S15 and Notes S1–S3

Document S2. Article plus supplemental information
