# Supplementary material for: Conductive dendrite engineering of single-crystalline two-dimensional dielectric memristors
Source: Innovation (Camb). 2025 Mar 18;6(6):100885. doi: 10.1016/j.xinn.2025.100885 (PMC12169237; doi:10.1016/j.xinn.2025.100885)
Supplement: Document S1. Figures S1–S15 and Notes S1–S3 [file mmc1.pdf]

The Innovation, Volume *6*

Supplemental Information

Conductive dendrite engineering of single-crystalline two-dimensional dielectric memristors

Yu Kang, Xingyu Zhai, Quan Yang, Baoshi Qiao, Zheng Bian, Haohan Chen, Huan Hu, Yang Xu, Ming Tian, Neng Wan, Wenchao Chen, Yang Chai, Yuda Zhao, and Bin Yu

Supplemental Information

The PDF file includes:

Note S1 Characterization of SJEM.

Note S2 Dynamics of silver ions migration modeling.

Note S3 The model of retention in h-BN memristor with various I_{cc}.

Figs. S1 to S15

Supplemental Notes

Supplemental Note 1 | Characterization of SJEM

Previous reports have the proposed visualization of conductive filaments based on transmission electron microscopy (TEM)[1,2] and conductive atomic force microscopy (C-AFM)[3,4]. These methods provide insights into the physiochemistry of conductive path. However, these destructive and invasive characterization methods give additional difficulties to sample preparation and characterization. CAFM is unable to characterize curved conductive filaments, while TEM faces challenges in analyzing multiple conductive filaments over a larger area.

Scanning Joule expansion microscope (SJEM) is an AFM-based non-invasive characterization tool that uses an AFM probe in physical contact with an electrode to measure the height difference in thermal expansion of the electrode surface[5]. E. Puyoo and D. Albertini used SJEM to detect conductive filaments in memristors firstly, demonstrating that this technique can non-destructively locate conductive filaments within memristors[6]. In this study, SJEM was performed using a standard AFM (Olympus AC240TSA-R3) combined with an external signal generator (SIGLENT SDG2122X) and a lock-in amplifier (Sine Scientific). Figure S10a illustrates the basic working principle of SJEM. The expansion signal on the electrode surface comes from the Joule heat. The high current density passes through the h-BN layer, generating heat dissipation and surface expansion when device operating in the LRS state. This can be detected by monitoring the deflection of AFM contact mode probe. The surface displacement images were obtained by a signal recovery OE1022 lock-in amplifier, which analyzes the deflection signal of the AFM four-quadrant photodiode.

Applying a pulsed voltage using a signal generator to the device will generate Joule heat inside the h-BN layer, and the heating effect can cause a slight expansion of the top Ag electrode surface. The dissipated Joule power (P_{Joule}) can be expressed as:

$$P_{Joule} \propto I^2 R_{LRS}$$

The tiny thermal expansion will cause the AFM cantilever to bend, which is amplified by a laser and detected by a four-quadrant photodiode. The output signal from the photodetector is then demodulated by a lock-in amplifier (Sine Scientific OE1022) to ultimately obtain the surface deformation signal. In this process, the AFM operates in contact mode, where the probe (Olympus AC240TSA-R3) maintains a constant contact force with the surface, ensuring that the thermal expansion signal is effectively transferred to the cantilever through the probe. Notably, the modulation frequency in this study is significantly higher than the AFM feedback cutoff frequency (1.5 kHz), ensuring that thermal expansion does not interfere with AFM topography measurements.

Figure S11b shows the noise image of the SJEM system used in this study. The thermal expansion resolution obtained from the image is 0.4 pm (S/N = 1). The spatial resolution of the measurement can be estimated from the image using the following formula:

$$\Delta x = \frac{\Delta L}{\partial L/\partial x}$$

Where, ΔL represents the thermal expansion resolution, and $\partial L/\partial x$ corresponds to the maximum gradient in Figure 3b. The spatial resolution is calculated accordingly to be ~10 nm.

In the work, the vertically confined filament channel creates highly localized thermal expansion points (n_{SV} = 6.5×10^{13} cm^{-2}), while dendritic filaments expand the thermal expansion points due to the heat generated by "branching" (n_{SV} = 2.0×10^{13} cm^{-2}). In addition, the presence of multiple conductive paths leads to multiple thermal expansion points (n_{SV} = 9.4×10^{13} cm^{-2}). Besides, the memristor with n_{SV} of 6.5×10^{13} cm^{-2} shows smallest LRS resistance when all the devices have the same I_{cc}, therefore displaying the smallest height change compared to the other devices (height $\propto P_{Joule} \propto I_{cc}^2 R_{LRS}$). The results prove that local thermal expansion formed during the operation of the device is consistent with I-V characterizations.

Supplemental Note 2 | Dynamics of silver ions migration modeling

A compact model is developed to explain the influence of SV defect densities on the electrical behavior of memristor, which is applicable when n_{SV} is not enough to form large-size vacancies. The proposed model considers the variation of Ag ions hopping distance, the different conductive mechanisms in HRS and LRS, and the dynamic percentage of Ag-occupied vacancies (*P*, which is defined as the number of occupied vacancies divided by the total number of vacancies).

During the set process, the oxidized Ag ions gradually fill into the vacancies, leading to the increased percentage (ΔP_+) of Ag-occupied vacancies in the h-BN layer, which was described as,

$$\Delta P_+ = \Delta t \cdot v_0 \cdot exp\left(-\frac{E_a-\gamma ZqE}{k_B T}\right)(1-P) \tag{1}$$

where *P* is the percentage denoting the number of occupied vacancies divided by the total number of vacancies, v_0 is the fitting parameter proportional to the vibration frequency of Ag^+ (*f*), E_a is the activation energy of Ag^+, γ is the electric field enhancement factor, Z is the charge number, *q* is the unit charge, *E* is the electric field, k_B is the Boltzmann constant, *T* is the average temperature of memristor.

During the reset process, the Ag ions hop back from the vacancies towards the Ag electrode, resulting in the decreased *P* and the reduced percentage of Ag-occupied vacancies (ΔP_-), and there is,

$$\Delta P_- = \Delta t \cdot v_0 \cdot exp\left(-\frac{E_a-\gamma ZqE}{k_B T}\right) \cdot P \tag{2}$$

The temperature of the device (*T*) is calculated by an equivalent thermal resistance model, as the following formula,

$$T = T_0 + IVR_{th} \tag{3}$$

where T_0 is the ambient temperature and R_{th} is the effective thermal resistance.

The dominant conduction mechanism for LRS is hopping conduction. The Ag^+ transport between vacancies is hopping, and the hopping probability P_{hop} can be calculated by the Mott hopping model, and there is,

$$P_{hop} = f \cdot exp\left(-2al - \frac{W-e\lambda_{hop}E}{k_B T}\right) \tag{4}$$

where *a* is the attenuation length of Ag^+ wave-function, *l* is the hopping distance ($l \sim \sqrt[3]{\frac{1}{d_{SV-3D}}}$, $d_{SV\text{-}3D}$ is the single vacancy density of the whole h-BN layer), *W* is the energy separation of two vacancies, and λ_{hop} is the electric field enhancement factor. According to hopping model, the threshold electric field (E_{min}) to causes the resistive switching of a certain device is linearly proportional to the hopping distance *l* for the certain P_{hop}. When $E < E_{min}$, γ is set as 0.

When the memristor is in HRS, the thermionic emission is regarded as the major dominant conduction mechanism because of the weak correlation between current density and electric field and the hopping conduction dominates the LRS conduction, which was defined as,

$$J = J_0 exp\left(\left(-\frac{p}{P_0}\right)^{-\frac{1}{3}}\right) sinh(\frac{V}{V_0}) + A_G T^2\, exp\left(-\frac{W_{th} - \sqrt{\lambda_{th} E}}{k_B T}\right) \tag{5}$$

where J_0, P_0, V_0 are characteristic current, percentage, and voltage, A_G is a material-specific correction factor, W_{th} is barrier height for an escaping Fermi-level electron, λ_{th} is the electric field enhancement factor.

Supplemental Note 3 | The model of retention in h-BN memristor with various I_{cc}.

Taking the average of the device resistance at 500 s intervals, we find that the resistance decreases slowly between 10^3 s and 10^4 s at the I_{cc} of 10 μA to 10 nA. The resistance change rate during the retention time and the activation energy of silver ion diffusion are combined to establish a corresponding model, which can predict longer retention times.

In this model, we believe that the redistribution of silver ions in the h-BN layer is responsible for the resistance change. The distribution of silver ions in h-BN has two parts: a low-occupancy region and a high-occupancy region when the device is in LRS. Then silver ions in high-occupancy region will be redistributed and diffuse to low occupancy areas under repeated voltage stimulation. This is the main reason for the slight drop of resistance. Finally, the silver ions are evenly distributed in the h-BN layer, and the resistance becomes stable. Storage states of the device during periods of 10 years and beyond are distinct and stable. And the retention behaviors could be written as,

$$\frac{dP}{dt} = \eta(P_{h0} - P_{l0})\left(1 - exp\left(-\frac{t}{\tau}\right)^{\frac{1}{\sqrt{2}}}\right) \quad (6)$$

$$\tau = \beta\, exp\left(\frac{E_a}{k_B T}\right) \quad (7)$$

η, β are the scaling parameters, P_{h0} is the initial occupancy of the high-occupancy region, and P_{l0} is the initial occupancy of the low-occupancy region. In fact, the memristor has good retention performance even over longer times due to the strong interaction of Ag atoms with neighboring h-BN atoms in the confined conduction paths.

Supplemental Figures

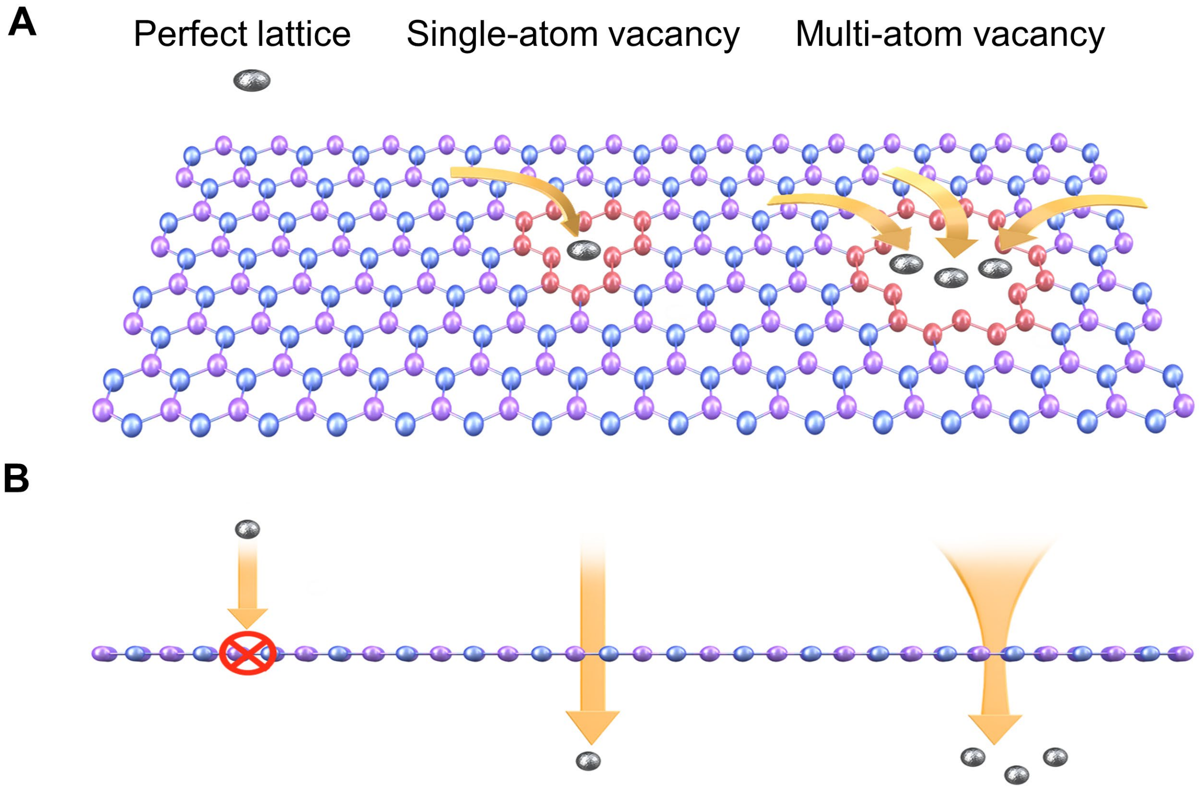

Figure S1. Impermeability of h-BN and selective permeability via defective sites. A. Schematic of Ag atoms migration through h-BN monolayer. Left: Ag^{+} cannot pass through h-BN monolayer with perfect lattice. Middle and right: the Ag^{+} migration is limited by the size of defective nanopore (single-atom vacancy or multi-atom vacancy) in the monolayer h-BN. B. Schematic diagram of three situations with the cross-sectional view.

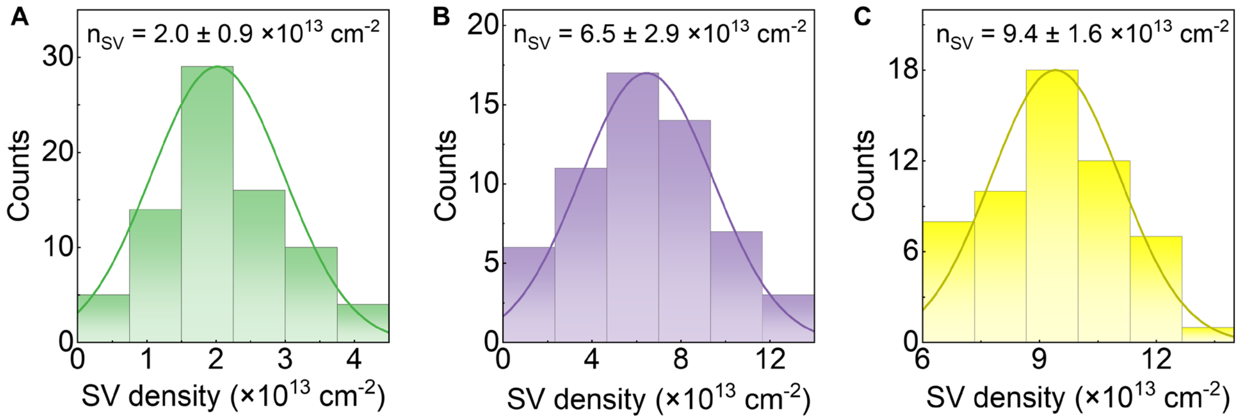

Figure S2. Characterization of defect density in single-crystalline h-BN. (A-C) The single vacancy (SV) defect density in single-crystalline h-BN corresponding to Figure 1g-i. The density was obtained by counting the number of SV defects in an area of 5×5 nm^2. The tunable defect density ranges from $2.0 \pm 0.9 \times 10^{13}$ cm^{-2} to $9.4 \pm 1.6 \times 10^{13}$ cm^{-2}.

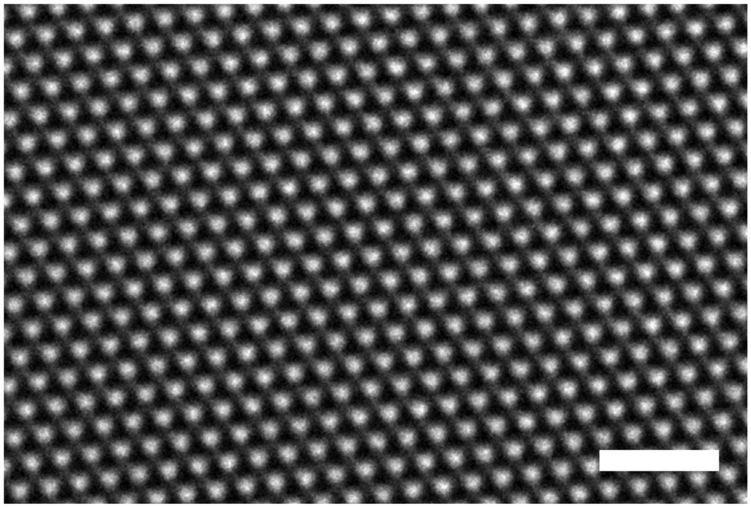

n_{SV} = 1.0 ×10^{10} cm^{-2}

Figure S3. The HRTEM image of the highest-quality h-BN with the lowest SV defect density. The SV density has been regarded as 1.0 ×10^{10} cm^{-2} in this paper. The scale bar is 1 nm.

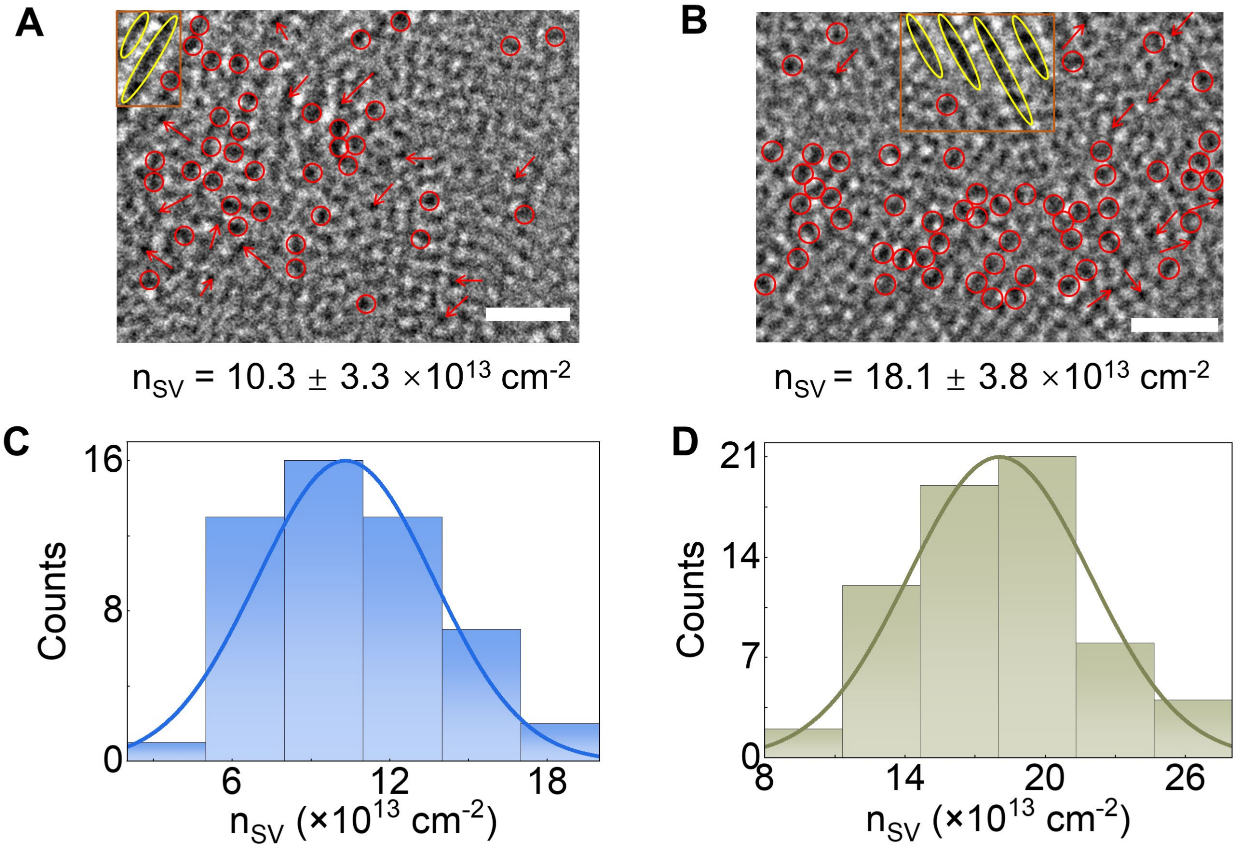

Figure S4. **The Characterization of defects in CVD-grown h-BN layers.** The CVD-grown h-BN memristor with high defect density and multiple defect types can act as the control device. The electrical characterizations (Fig. S8 and S9) demonstrate that several conductive filaments exist in the switching medium and the rupture of conductive paths in the CVD-grown h-BN layer requires high energy. (A-B) The HRTEM images of CVD-grown h-BN. The scale bar is 1 nm. (C-D) SV defect densities in Fig. S5a and S5b are 1.0 and 1.8×10^{14} cm^{-2}, respectively.

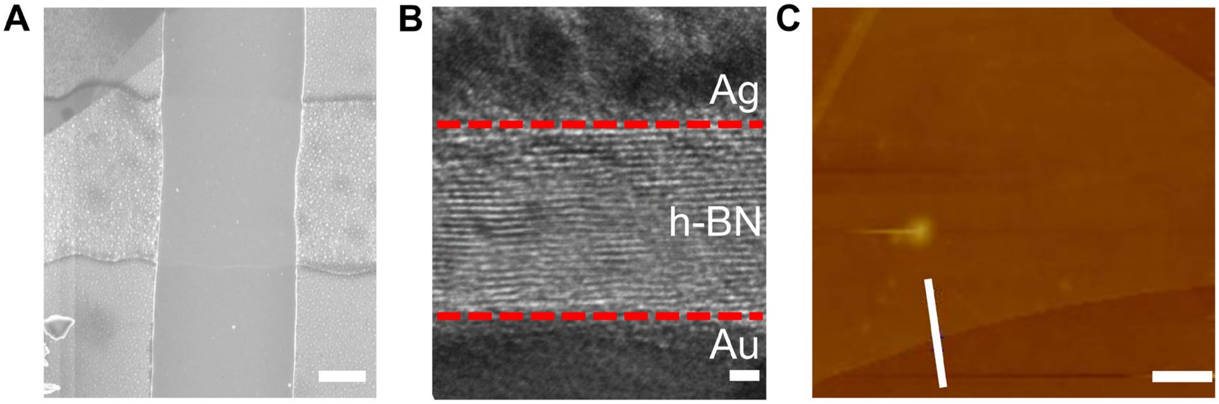

Figure S5. Device structure and material characterization. A. SEM image of the memristor device (scale bar is 1 μm). B. The cross-section TEM image showing the layer structure of single-crystalline h-BN (scale bar is 1 nm). C. The AFM image of single-crystalline h-BN, displaying the physical thickness of 6 nm (scale bar is 2 μm).

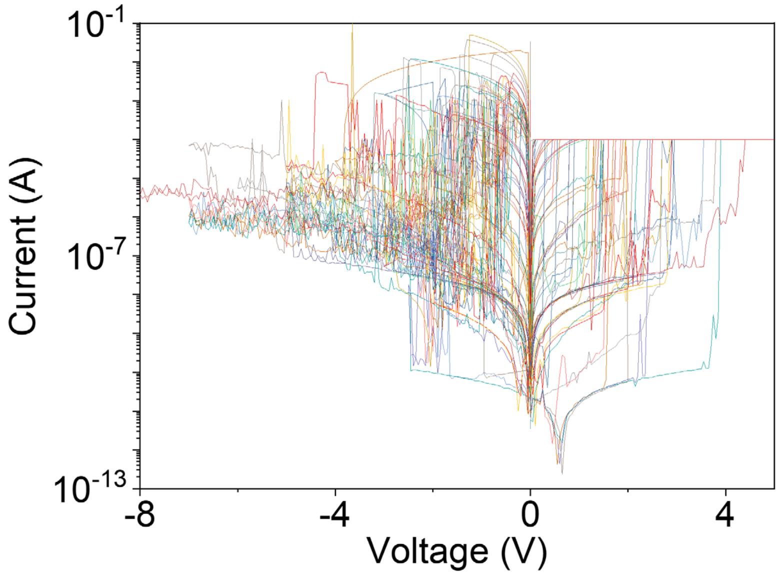

Figure S6. The performance of single-crystalline h-BN based memristor with SV density of 1.0 ×10^{10} cm^{-2}. The 100 DC I-V sweep curves were collected in single-crystalline h-BN based non-volatile memristor.

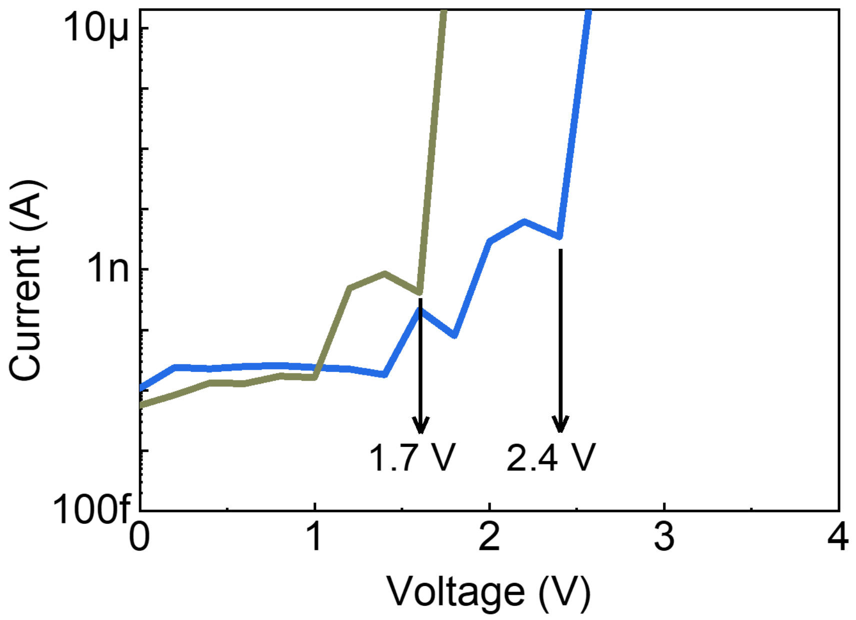

Figure S7. **The Forming Performance of CVD-grown h-BN based memristor.** The forming voltages of 1.7 V and 2.4 V correspond to $n_{SV}= 1.0\times10^{14}$ cm^{-2} and $n_{SV}= 1.8\times10^{14}$ cm^{-2} in CVD-grown h-BN, respectively.

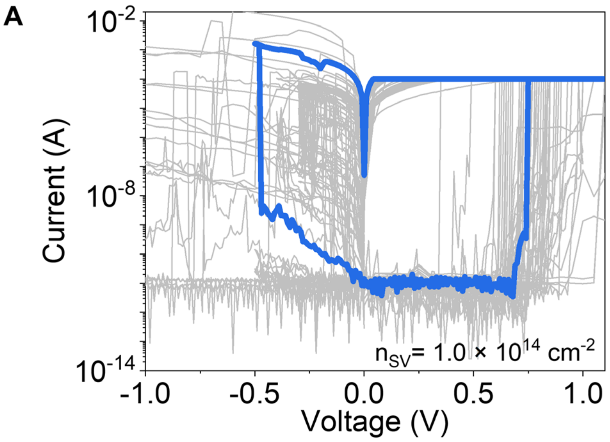

Figure S8. The resistive switching performance of CVD-grown h-BN based memristor. The measured 100 DC I-V sweeping curve of CVD grown h-BN based non-volatile memristor and A. $n_{SV} = 1.0\times10^{14}$ cm^{-2} and B. $n_{SV} = 1.8\times10^{14}$ cm^{-2}. The electrical characterizations demonstrate that several conductive filaments exist in the switching medium and the rupture of conductive paths in the CVD-grown h-BN layer requires high energy.

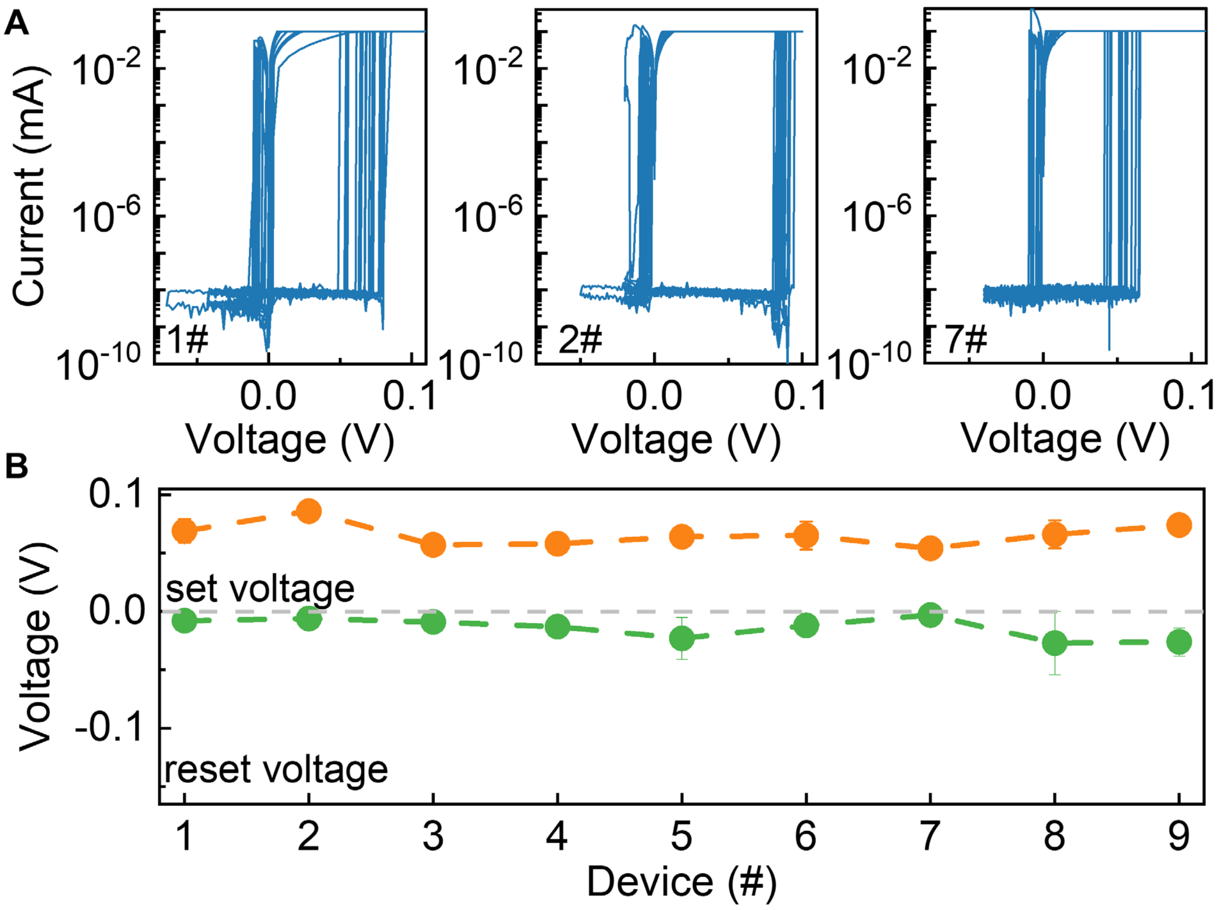

Figure S9. Device-to-device variation of single-crystalline h-BN based memristor with n_{SV} of 6.5 ×10^{13} cm^{-2}. A. Representative current versus voltage plots collected for different single-crystalline h-BN memristor with an active device size of 3 μm × 3 μm. B. Distribution of operating voltages in different devices.

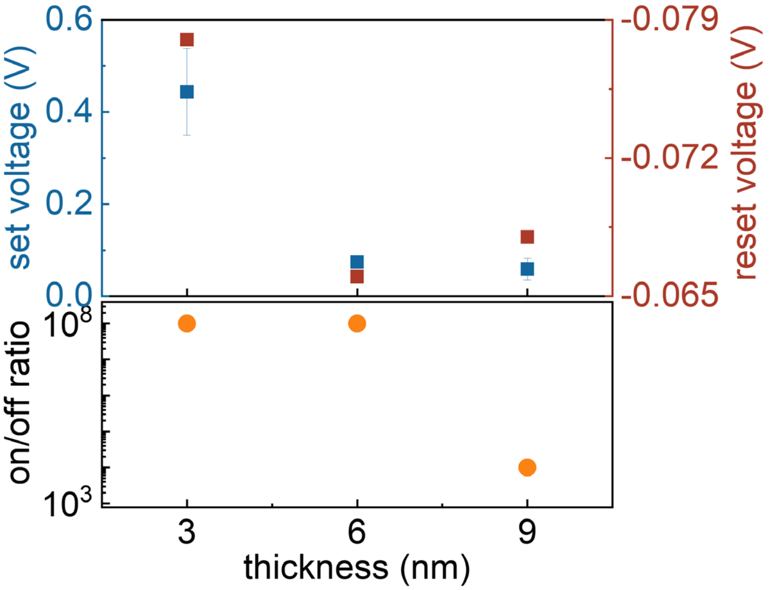

Figure S10. Dependence of operation voltage and resistance on/off ratio on single-crystalline h-BN based memristor with n_{SV} of 6.5 $\times 10^{13}$ cm^{-2}.

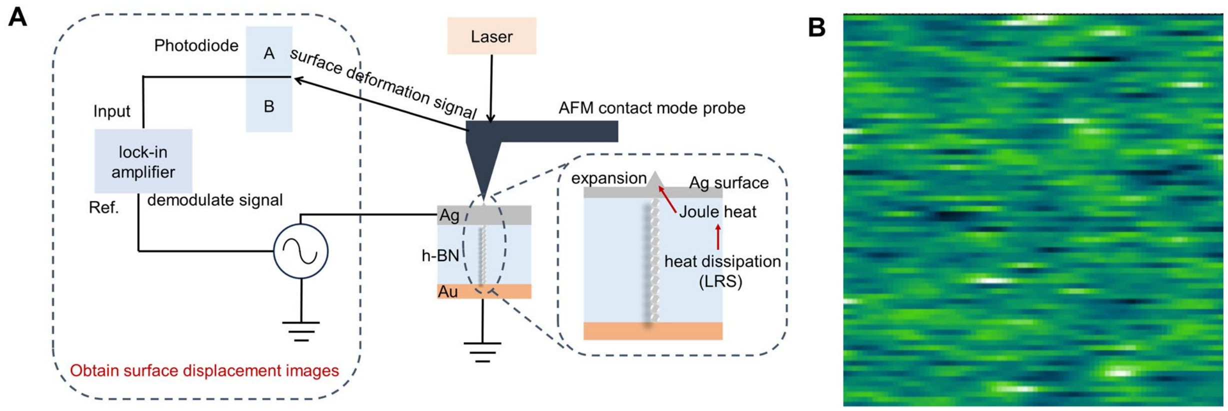

Figure S11. A. Schematic diagram of the experimental setup used for SJEM. B. The SJEM signal obtained in high resistance state of $n_{SV} = 6.5 \times 10^{13}$ cm^{-2}.

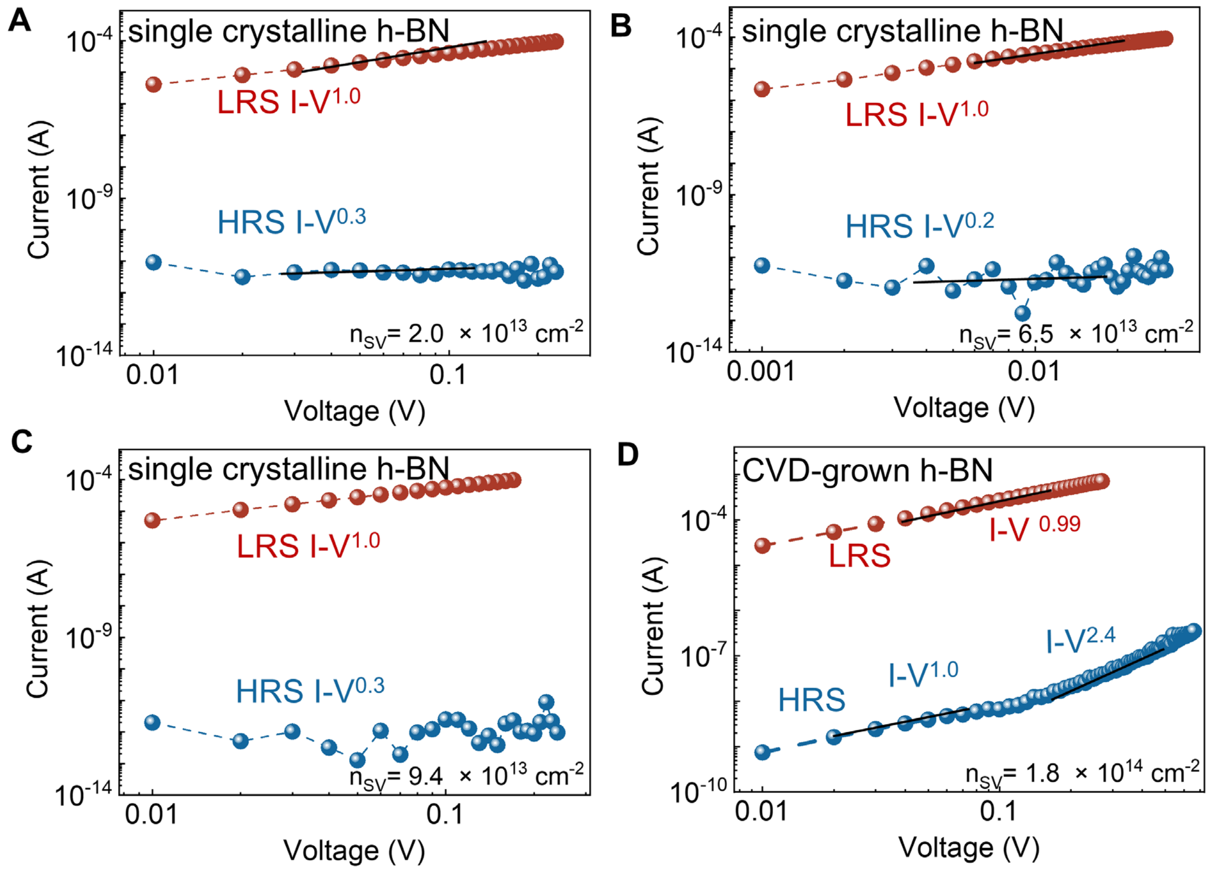

Figure S12. Demonstration of conductive dendrites engineered in single-crystalline h-BN based memristor. (A-D) The double logarithmic scales of h-BN memristor with n_{SV} of 2.0 $\times 10^{13}$ cm^{-2} to 1.8 $\times 10^{14}$ cm^{-2}.

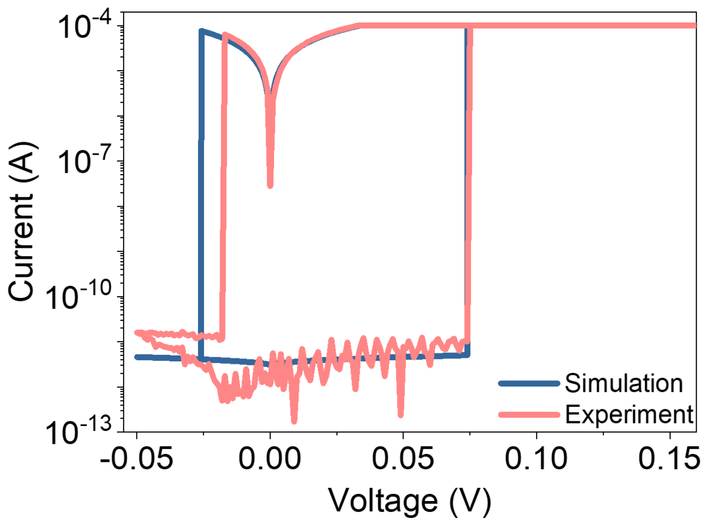

Figure S13. The simulated DC I-V curve of the h-BN memristor with n_{SV}= 6.5 × 10^{13} cm^{-2}. The viability of hopping model has been verified through the comparison between experiment data and simulation data.

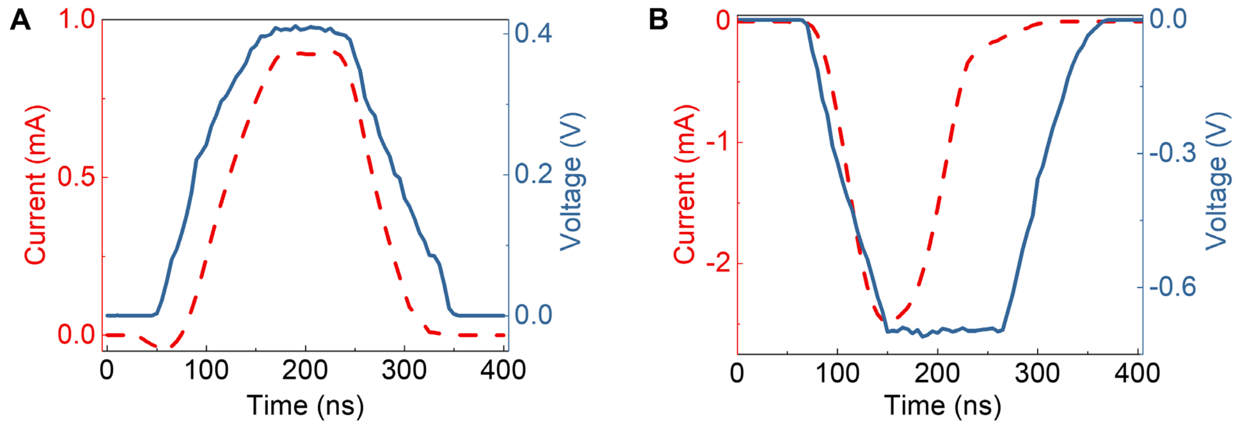

Figure S14. Performance of h-BN memristor under pulsed voltages. The detailed pulse operation of h-BN memristor. The time dependent current curves by applying A. SET voltage pulse and B. RESET voltage pulse.

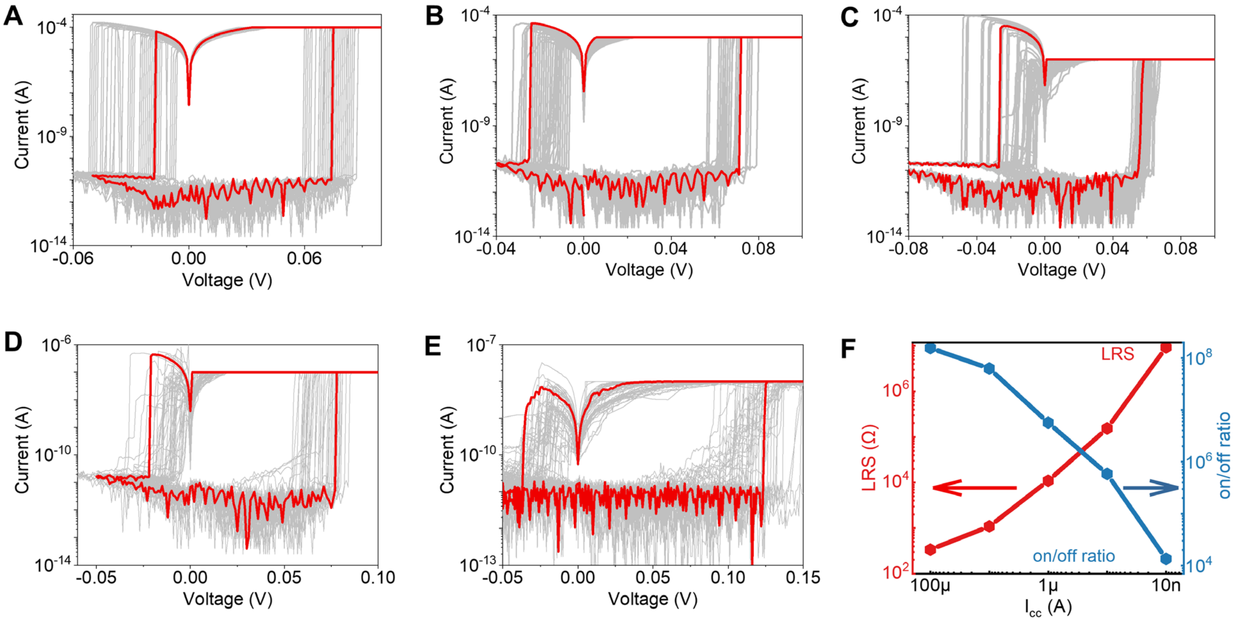

Figure S15. Performance of h-BN memristor under various I_{cc}. Non-volatile bipolar resistive switching for single-crystalline h-BN memristor with $n_{SV}=6.5\times10^{13}$ cm^{-2} when I_{cc} varies from A-E. 0.1mA to 10nA. F. Multiple LRS resistances and the corresponding on/off ratio fitted from 50 DC I-V cycles with varied I_{cc} (10 nA to 0.1 mA) in h-BN memristor with $n_{SV} = 6.5\times10^{13}$ cm^{-2}.

References

1. Yang, Y., Gao, P., Gaba, S., Chang, T., Pan, X., and Lu, W. (2012). Observation of conducting filament growth in nanoscale resistive memories. Nat Commun ***3***, 732. 10.1038/ncomms1737.
2. Li, Y., Loh, L., Li, S., Chen, L., Li, B., Bosman, M., and Ang, K.-W. (2021). Anomalous resistive switching in memristors based on two-dimensional palladium diselenide using heterophase grain boundaries. Nat. Electron. ***4***, 348-356. 10.1038/s41928-021-00573-1.
3. Chen, S., Mahmoodi, M.R., Shi, Y., Mahata, C., Yuan, B., Liang, X., Wen, C., Hui, F., Akinwande, D., Strukov, D.B., and Lanza, M. (2020). Wafer-scale integration of two-dimensional materials in high-density memristive crossbar arrays for artificial neural networks. Nat. Electron. ***3***, 638-645. 10.1038/s41928-020-00473-w.
4. Ranjan, A., Raghavan, N., O'Shea, S.J., Mei, S., Bosman, M., Shubhakar, K., and Pey, K.L. (2018). Conductive Atomic Force Microscope Study of Bipolar and Threshold Resistive Switching in 2D Hexagonal Boron Nitride Films. Sci Rep ***8***, 2854. 10.1038/s41598-018-21138-x.
5. Varesi, J., and Majumdar, A. (1998). Scanning Joule expansion microscopy at nanometer scales. Appl. Phys. Lett. ***72***, 37-39. 10.1063/1.120638.
6. Puyoo, E., and Albertini, D. (2020). Conductive Filament Localization Within Crossbar Resistive Memories by Scanning Joule Expansion Microscopy. IEEE Electron Device Lett. ***41***, 848-851. 10.1109/led.2020.2986543.
